# Geometrical tuning art for entirely subwavelength grating waveguide based integrated photonics circuits

**DOI:** 10.1038/srep24106

**Published:** 2016-05-05

**Authors:** Zheng Wang, Xiaochuan Xu, Donglei Fan, Yaguo Wang, Harish Subbaraman, Ray T. Chen

**Affiliations:** 1 Materials Science and Engineering Program, Texas Materials Institute, The University of Texas at Austin, Austin, Texas 78712, USA; 2 Department of Electrical and Computer Engineering, The University of Texas at Austin, 10100 Burnet Rd., MER 160, Austin, Texas 78758, USA; 3 Omega Optics, Inc., 8500 Shoal Creek Blvd., Bldg. 4, Suite 200, Austin, TX 78757, USA; 4 Department of Mechanical Engineering, The University of Texas at Austin, Austin, Texas 78712, USA

## Abstract

Subwavelength grating (SWG) waveguide is an intriguing alternative to conventional optical waveguides due to the extra degree of freedom it offers in tuning a few important waveguide properties, such as dispersion and refractive index. Devices based on SWG waveguides have demonstrated impressive performances compared to conventional waveguides. However, the high loss of SWG waveguide bends jeopardizes their applications in integrated photonic circuits. In this work, we propose a geometrical tuning art, which realizes a pre-distorted refractive index profile in SWG waveguide bends. The pre-distorted refractive index profile can effectively reduce the mode mismatch and radiation loss simultaneously, thus significantly reduce the bend loss. This geometry tuning art has been numerically optimized and experimentally demonstrated in present study. Through such tuning, the average insertion loss of a 5 μm SWG waveguide bend is reduced drastically from 5.43 dB to 1.10 dB per 90° bend for quasi-TE polarization. In the future, the proposed scheme will be utilized to enhance performance of a wide range of SWG waveguide based photonics devices.

Silicon photonics has been attracting tremendous interests in the last decade^1^ due to its great potential in realizing low cost photonic chips through the use of well-established CMOS manufacturing technology^2^. However, the fact that silicon does not possess a direct band gap or second-order nonlinearity makes it challenging to generate or control photons. Hybrid integration of silicon and functional cladding materials has been considered as a potential solution to this issue^3^. Several structures, such as slot waveguides^4^,^5^,^6^ and photonic crystal waveguides^7^,^8^,^9^, have been exploited to enhance the light-matter interaction. Subwavelength grating (SWG) waveguide, comprising of a periodic arrangement of high and low refractive index materials with a pitch less than one wavelength, has received considerable attention in recent years^10^,^11^,^12^,^13^,^14^,^15^,^16^,^17^,^18^,^19^,^20^,^21^. Bloch modes can be supported by this periodic arrangement of silicon pillars and cladding material, and therefore in theory photons can propagate without being attenuated by the discontinuity of media^10^,^11^. Furthermore, SWG provides another degree of freedom to precisely control a few important waveguide properties, such as refractive index^12^,^13^,^14^, dispersion^15^, and mode overlap volume^16^, which are determined by materials constituting the waveguides, as demonstrated previously. The control of these properties enables significant improvements in performance over conventional waveguide based devices, such as grating couplers^12^,^15^, directional couplers^17^ , sensors^18^, filters^19^ and modulators^20^. However, the practical applications of SWG waveguide have been greatly compromised by the large loss of SWG waveguide bends. For instance, a 10 μm radius 90° bend has an insertion loss of ~1.5 dB^21^. To avoid the significant loss introduced by SWG waveguide bends, a common approach is to taper the SWG waveguide to conventional strip waveguides at the bend, and further tapering back to SWG waveguides afterwards^11^. Although the strip waveguide bends can reduce the loss, the taper adds additional loss and wastes the precious silicon chip surface^21^. Therefore, to achieve the goal of building integrated photonics system with entirely SWG waveguides, it is critical to design an innovative SWG waveguide bend with low-loss and small radius. In this work, we report a completely new type of SWG waveguide bends with substantially reduced loss via optimizing the refractive index profile with strategically tuned silicon pillar geometries.

## Result

### Theoretical analysis of losses in SWG waveguide bends

**Figure 1:**
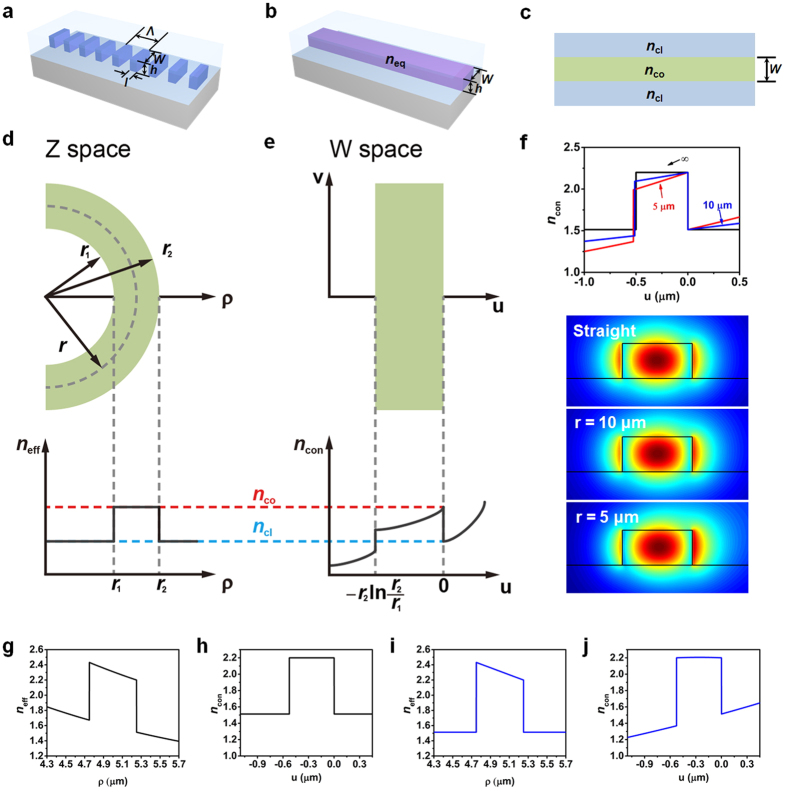
Theoretical analysis of bend losses in SWG waveguides and pre-distortion compensation method. (**a**) 3D schematic of a SWG waveguide. (**b**) 3D schematic of a SWG waveguide approximated via EMT. (**c**) Schematic of the final approximated result of a SWG waveguide. (**d**) Geometry and effective refractive index profile of a SWG waveguide in Z space. (**e**) Geometry and effective refractive index profile of a SWG waveguide in W space. (**f**) Conformal refractive index profiles in W space with different bend radius *r* and corresponding mode profiles. (**g**) The ideal effective refractive index profile in W space. (**h**) The conformal transformation result of (**g**) (in Z space). (**i**) The effective refractive index profile in W space via linear pre-distortion compensation method. (**j**) The conformal transformation result of (**i**) (in Z space). (**g–i**) Represent *r* = 5 μm cases.

Figure 1a shows a 3D schematic of a typical SWG waveguide, where *Λ* is the period of the SWG structure, *l*, *w*, and *h* are the length, width, and height of silicon pillars, respectively. In our simulations and experiments, SU-8 (*n* = 1.58) is selected as the top cladding material. The period *Λ* is 300 nm. A typical silicon pillar (duty cycle equals to 0.5) has a geometry of *l* × *w* × *h* = 150 nm × 500 nm × 250 nm without further optimization^11^. To simplify the analysis, two approximations are assumed sequentially without losing generality. Firstly, SWG waveguide is approximated as a uniform strip waveguide with an equivalent refractive index (*n*_eq_) shown in Fig. 1b. The equivalent refractive index *n*_eq_ = 2.70 is calculated by using the first order effective medium theory (EMT)^12^. Secondly, the 3D strip waveguide is further simplified into a 2D waveguide through effective refractive index approximation, as shown in Fig. 1c 22. The effective refractive indices of cladding and core are *n*_cl_ = 1.51 and *n*_co_ = 2.20, respectively (see Supplementary Fig. S1). Figure 1d shows the geometry and effective index profile of a SWG waveguide bend after the aforementioned two approximations in Z space (2D complex plane, *Z* = *x* + i*y*) with cylindrical coordinates. The effective refractive index profile can be expressed as:


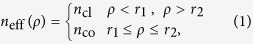


where *n*_cl_ and *n*_co_ are the effective refractive index of the cladding (*n*_cl_ = 1.51) and core of a waveguide (*n*_co_ = 2.20), respectively, and *r*_1_ and *r*_2_ denote the radius of the inner and outer edge, respectively. To unveil the source of bend loss, conformal transformation method, which has been widely used to analyze conventional strip waveguide bends^22^,^23^,^24^,^25^,^26^, is applied. By using the conformal transformation *W* = *r*_2_ln*Z*/*r*_2_, a bend in Z space can be taken equivalently as a straight waveguide in W space (2D complex plane, *W* = *u* + i*v*)^23^. Figure 1e shows the geometry and conformal refractive index profile in W space with transformed Cartesian coordinate system. The first order approximation of transformed effective refractive index (here we name it conformal refractive index to eliminate ambiguity) *n*_con_(*u*) can be calculated by^24^


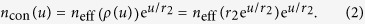


Figure 1f shows *n*_con_(*u*) with different bend radius *r* and corresponding mode profiles. The black curve represents the conformal transformation (in W space) of a straight waveguide, which can be treated as a bend with infinite radius *r* = ∞. With the decrease of bend radius, the refractive index profile becomes asymmetric and tilted, which shifts the mode to the outer edge (right side) of an SWG waveguide bend, as shown by the mode profiles (simulated via FIMMWAVE, a commercially available software developed by PhotonDesign) in Fig. 1f. The delocalization of optical modes due to bending leads to a mode mismatch between straight and curved waveguide segments and an increase in radiation loss. To quantify the change of refractive index profile induced by a bend, we define a distortion factor *D* as


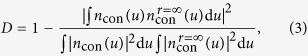


where 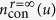 is the conformal refractive index profile in W space when the bend radius is infinite, as shown by the black plot in Fig. 1f. The refractive index distortion factor *D* increases with the decrease of the bend radius (see Supplementary Fig. S2). When the bend radius *r* > 5 μm, *D* is less than 1% and insensitive to the bend radius. Thus, we choose to study the case of *r* = 5 μm.

### Pre-distortion compensation method

To eliminate the distortion after conformal transformation, ideally the conformal refractive index profile of a bend in W space needs to satisfy the condition of 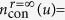 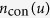, which is extremely difficult to achieve due to the limited control of the refractive index profile of conventional strip waveguides. SWG waveguides, however, provide the freedom for tailoring the refractive index profile and minimizing *D*. According to Eq. (2), 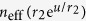 should be equivalent to 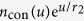 to cancel the *u* dependent term. Thus, the idea effective refractive index profile in Z space should follow the equation as below


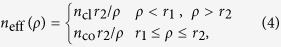


This ideal effective refractive index profile in Z space is shown in Fig. 1g and the result of conformal transformation in W space is shown in Fig. 1h (r = 5 μm). Even though the refractive index of the cladding is not tunable, since the majority of mode is concentrated in the high index region, the distortion due to the cladding refractive index can be safely ignored. According to Eq. (4), *n*_eff_(*r*_2_) = *n*_co_ and *n*_eff_(*r*_1_) = *n*_co_*r*_2_/*r*_1_. Therefore, *n*_eff_(*r*_2_) and *n*_eff_(*r*_1_) equal to 2.43 and 2.20, respectively. Considering that it is not realistic to fabricate a SWG waveguide bend with the effective refractive index profile described by Eq. (4) and knowing *r* ≫ *w*, we took a linear approximation and simplified the equation to


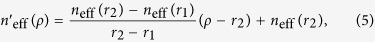


where 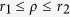. Figure 1i illustrates the effective refractive index profile in Z space via this linear pre-distortion compensation method. The refractive index profile after conformal transformation is shown in Fig. 1j (in W space). Validation of this approximation can be found in Supplementary Fig. S3.

### Trapezoidal silicon pillar design and optimization

**Figure 2:**
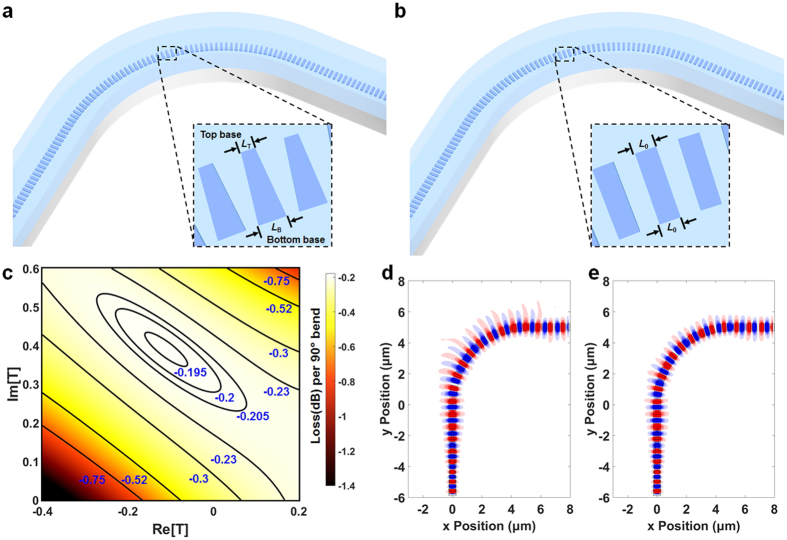
Design and optimization of the geometrical tuning art. (**a**) 3D schematic of an SWG waveguide bend built with trapezoidal silicon pillars. (**b**) 3D schematic of an SWG waveguide bend built with conventional rectangular silicon pillars. (**c**) Contour plot of bend loss for complex turning factors. (**d**) non-tuned(T = 0, rectangular silicon pillars) and (**e**) optimally tuned (T = −0.067 + i0.4, trapezoidal silicon pillar with 140 nm top base and 210 nm bottom base) SWG waveguide bends via 3D FDTD simulation.

To implement the proposed approach into SWG waveguide bends, trapezoidal silicon pillars are exploited to create the asymmetric effective index profile. The schematic of trapezoidal silicon pillars is shown in Fig. 2a and the schematic of conventional rectangular silicon pillars is shown in Fig. 2b. For trapezoidal silicon pillars, the top base (at the outer edge) and bottom base (at the inner edge) can be tuned to minimize the bend loss. To quantify this tuning process, we define a complex tuning factor as


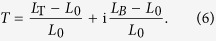


Here *L*_T_ and *L*_B_ are the widths of the top base and bottom base of the trapezoidal silicon pillars, respectively (see Fig. 2a). *L*_0_ is the width of a conventional rectangular silicon pillar (see Fig. 2b). To verify the aforementioned theoretical analysis, we used full 3D FDTD simulation to scan the tuning factor (simulated via FullWAVE^TM^, a commercially available software developed by Synopsys, Inc.). The simulation results are summarized in a contour plot (see Fig. 2c). It is found that a trapezoidal silicon pillar with a tuning factor of T = −0.067 + i0.4 (140 nm top base and 210 nm bottom base) offers a minimum bend loss of 0.192 dB per 90° bend. It is 50.1% of the bend loss of an SWG waveguide bend built with conventional rectangular silicon pillars (T = 0, 0.383 dB per 90° bend). The calculated effective indices of the optimized geometry of silicon pillars at the outer (2.11) and inner edges (2.63) agree well with the analytical results of 2.20 and 2.43, respectively. The discrepancy between the analytical analysis and the 3D FDTD is primarily caused by EMT and effective refractive index approximations. The optical field distributions of 90° SWG waveguide bends of 5 μm in radius without and with pre-distortion compensation are shown in Fig. 2d,e, respectively. With the optimized design of silicon pillars, the optical field is better confined in the waveguide region (Fig. 2e) compared with the conventional SWG waveguide with rectangular pillars (Fig. 2d).

### Device fabrication and characterization

**Figure 3:**
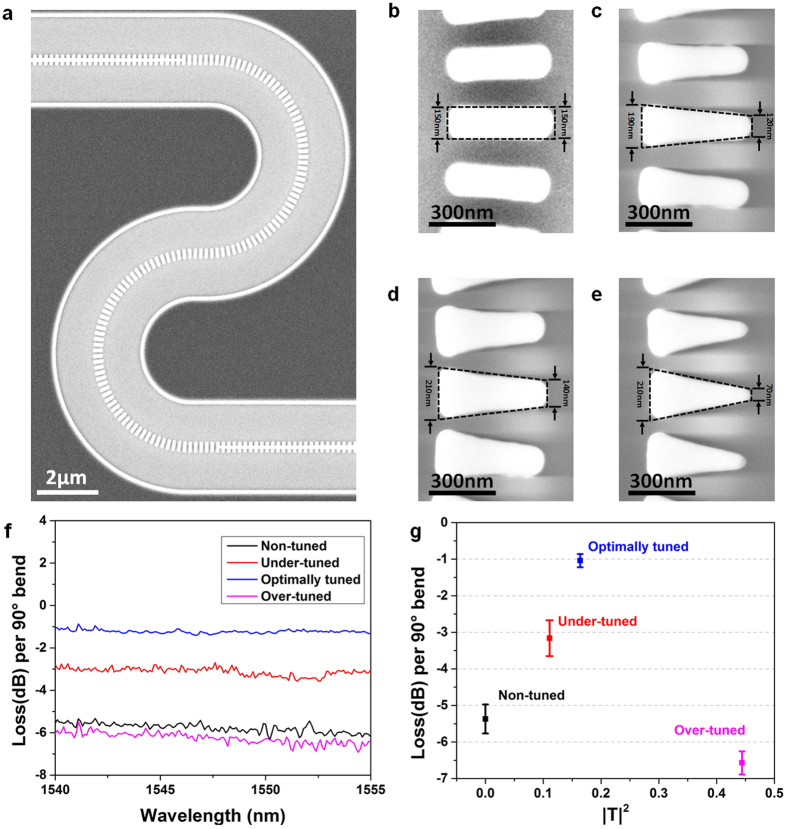
Experimental demonstration of the geometrical tuning art. (**a**) SEM image of a typical device. SEM images of (**b**) non-tuned silicon pillars (T = 0, |T|^2^ = 0), (**c**) under-tuned silicon pillars (T = −0.2 + i0.267, |T|^2^ = 0.111), (**d**) optimally tuned silicon pillars (T = −0.067 + i0.4, |T|^2^ = 0.164), and (**e**) over-tuned silicon pillars (T = −0.533 + i0.4, |T|^2^ = 0.444). (**f**) Transmission spectra of the four types of silicon pillars. (**g**) Statistical insertion loss of the four types of silicon pillars at 1550 nm.

Four types of silicon pillars: non-tuned rectangle (T = 0), under-tuned trapezoid (T = −0.2 + i0.267), optimally-tuned trapezoid (T = −0.067 + i0.4) and over-tuned trapezoid (T = −0.533 + i0.4) have been fabricated for demonstration (see Method section). The agreement of the morphology of the devices with the design is confirmed by scanning electron microscopy as shown in Fig. 3a. Each device has four 90° bends. Figure 3b–e show the high magnification SEM images of the four types of SWG waveguide bends: non-tuned, under-tuned, optimally tuned, and over-tuned. It is very challenging to fabricate the trapezoidal shape pillars precisely. The fabricated silicon pillars have round corners and curved sidewalls due to electron diffraction and charge accumulation. Pre-compensation has been made in the E-beam lithography pattern to ensure the desired pattern can be precisely transferred into silicon layer. To quantitatively evaluate the pre-compensation, we define a pattern transfer fidelity factor (PTFF). The details of PTFF can be found in Supplementary Note S1. Fabrication parameters have been carefully tuned to make the PTFF as close to 1 as possible. For our fabrication, the PTFFs are more than 0.95 at the resolution of SEM images, which is shown in Supplementary Table S1.

After spin-coating the SU-8 cladding, the devices are tested in a customized grating coupler alignment system employed in our previous work^12^,^14^,^27^ (see Method section and Supplementary Fig. S4). Figure 3f shows the transmission spectra of the four SWG waveguide bends between 1540 nm and 1555 nm for quasi-TE polarization. The insertion loss of the four representative bends at 1550 nm is shown in Fig. 3g, where the optimized trapezoidal silicon pillars is as low as 1.10 dB per 90° bend, only 20.3% of that of the non-tuned rectangular silicon pillars (5.43 dB per 90° bend). The details of measurements can be found in Methods section. Spectra of all devices with and without subtracting the grating couplers’ response from 1530 nm to 1580 nm can be found in Supplementary Figs S5 and S6, respectively. Compared to the earlier demonstration of 10 μm bend with a duty cycle of 0.8^21^, we are able to reduce the bend radius to 5 μm without increasing the insertion loss significantly. The loss can be further reduced if the duty cycle increases from 0.5 to 0.8. When the pillars are over-tuned, the refractive index distortion and mode mismatch increase, and thus the loss increases. Although the insertion loss of the bends are higher than that of the theoretical prediction, which could be attributed to the sidewall roughness of the silicon pillars, the experimental results clearly demonstrate that the loss of SWG bends can be significantly reduced by strategically optimizing the geometries of the silicon pillars.

## Discussion

In conclusion, via theoretical analysis, numerical simulation, and experimental demonstration, we have shown that through pre-distortion compensation with optimized trapezoidal silicon pillars, the insertion loss of SWG waveguide bends can be reduced substantially. Compared to conventional SWG waveguide bends based on rectangular silicon pillars, an average reduction of 79.7% of the insertion loss has been achieved experimentally via optimization of trapezoidal silicon pillars. When a 90° bend is inevitable in building complex photonics circuits, the conventional solutions^21^, including two adiabatic tapers and a strip waveguide based bend, requires 60 μm × 60 μm = 3,600 μm^2^ of the silicon surface while still have an insertion loss about ~1.4 dB (the bend loss and surface of the strip waveguide bend are ignored). With our approach, only 5 μm × 5 μm = 25 μm^2^ (7% of 3,600 μm^2^) of the silicon surface is needed and the insertion loss is 1.10 dB (78.5% of 1.4 dB). For other duty cycles, our geometrical tuning art still works. More details can be found in Supplementary Table S2. This study is essential towards achieving the holy grail of entirely SWG waveguide based optical devices and circuits.

## Methods

### Device Fabrication and characterization

The devices are fabricated on a SOI wafer (manufactured by Soitec.) consisting of a 250 nm thick top silicon layer and a 3 μm thick buried oxide layer. All structures are patterned in a single E-beam lithography (JEOL 6000 FSE) step at the nanofabrication facility at the University of Texas at Austin. The patterns are then transferred into the underneath silicon layer through reactive-ion-etching (PlasmaTherm 790). SU-8 2005(manufactured by MicroChem Corp.) is spin-coated at 3000 rpm to form a 5 μm thick top cladding. Overnight baking in an oven at 80 °C is applied to reflow the SU-8 for a tough infiltration^28^.

To measure the loss per bend, four 90° SWG waveguide bends are cascaded, as shown in Fig. 3a. The two ends of the bends are connected to 4 mm long strip waveguides with 40 μm long strip-SWG waveguide mode converters. Two SWG grating couplers are used to couple light into and out from the testing structure. The reference waveguide is formed by removing the cascade four 90° SWG waveguide bends. The loss per bend is extracted by subtracting the transmission of the reference waveguide from the testing structure and divided by four. We fabricated and tested twelve testing structures for the four different types of SWG waveguide bends (non-tuned, under-tuned, optimally tuned and over-tuned). Each type has been fabricated and tested three times. For operation wavelength of 1550 nm, the statistical analysis has been summarized in Fig. 3g.

### Experiment set-up

Light from a broadband amplified spontaneous emission (ASE) source (1510 nm–1630 nm) is input to the device using a customized grating coupler alignment system (see Supplementary Fig. S4). The input and output fibers are mounted on two 10° wedges, which sit on tilting stages for adjusting coupling angle. Two xyz stages are used to align the fibers to on-chip grating couplers. A camera mounted on another xyz stage is tilted at 45° angle to visually assist the alignment. After passing through the devices, light signal is collected by the output fiber, which is fed to an optical spectrum analyzer (OSA) to record the optical spectra.

## Additional Information

**How to cite this article**: Wang, Z. *et al.* Geometrical tuning art for entirely subwavelength grating waveguide based integrated photonics circuits. *Sci. Rep.* **6**, 24106; doi: 10.1038/srep24106 (2016).

## Supplementary Material

Supplementary Information
