# Supplementary material for: Geometrical tuning art for entirely subwavelength grating waveguide based integrated photonics circuits
Source: Sci Rep. 2016 May 5;6:24106. doi: 10.1038/srep24106 (PMC4857108; doi:10.1038/srep24106)
Supplement: Supplementary Information [file srep24106-s1.pdf]

Supplementary Information
Geometrical tuning art for entirely subwavelength grating waveguide based integrated photonics circuits

Zheng Wang[1, 2, ★], Xiaochuan Xu[3, ★], Donglei Fan[1, 4], Yaguo Wang[1, 4], Harish Subbaraman[3], & Ray T. Chen[1, 2, 3,*]

[1]*Materials Science and Engineering Program, Texas Materials Institute, The University of Texas at Austin, Austin, Texas 78712, USA*
[2]*Department of Electrical and Computer Engineering, The University of Texas at Austin, 10100 Burnet Rd., MER 160, Austin, Texas 78758, USA*
[3]*Omega Optics, Inc., 8500 Shoal Creek Blvd., Bldg. 4, Suite 200, Austin, TX 78757, USA*
[4]*Department of Mechanical Engineering, The University of Texas at Austin, Austin, Texas 78712, USA*
[★]*These authors equally contributed to this work*
**Corresponding author: chenrt@austin.utexas.edu*

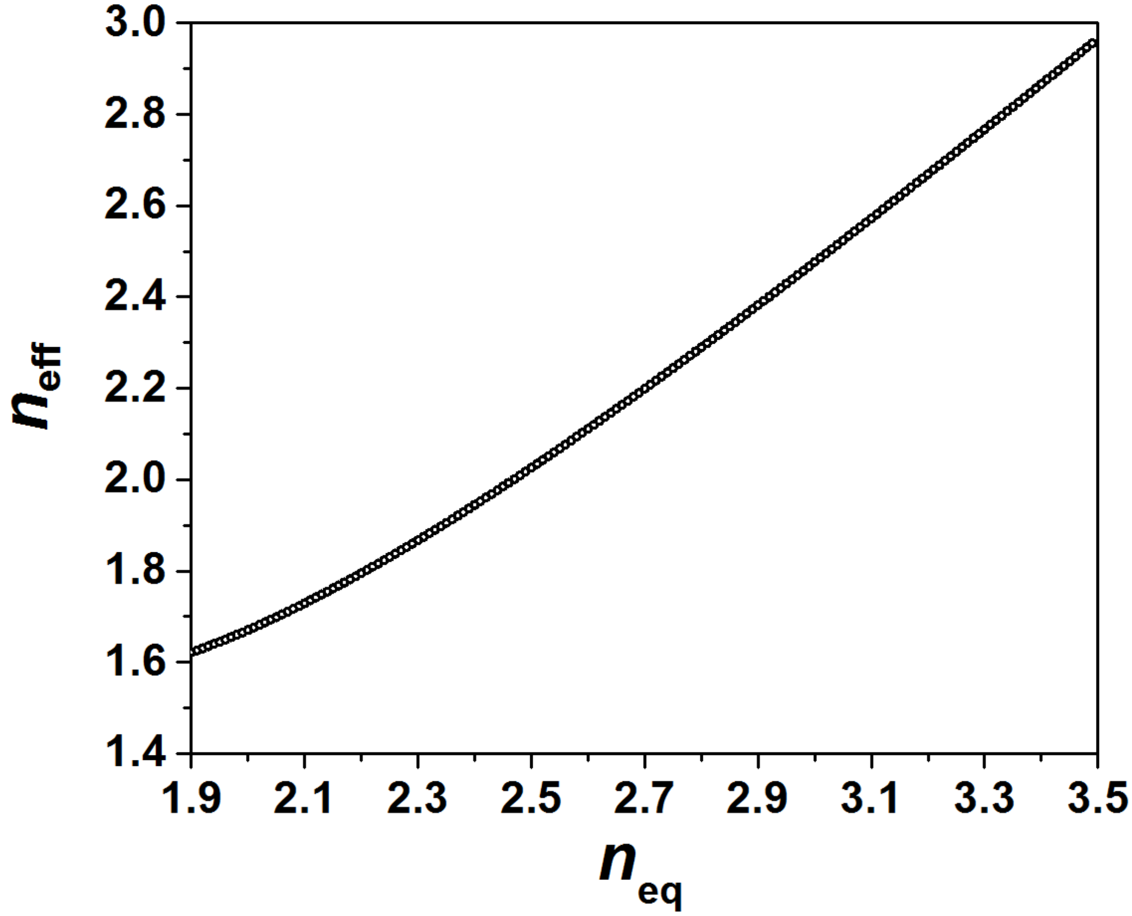

Supplementary Figure S1. The relationship between equivalent refractive index and effective refractive index. This relationship is simulated via CAMFR (CAvity Modelling FRamework, a freely available eigenmode expansion simulation tool http://camfr.sourceforge.net/).

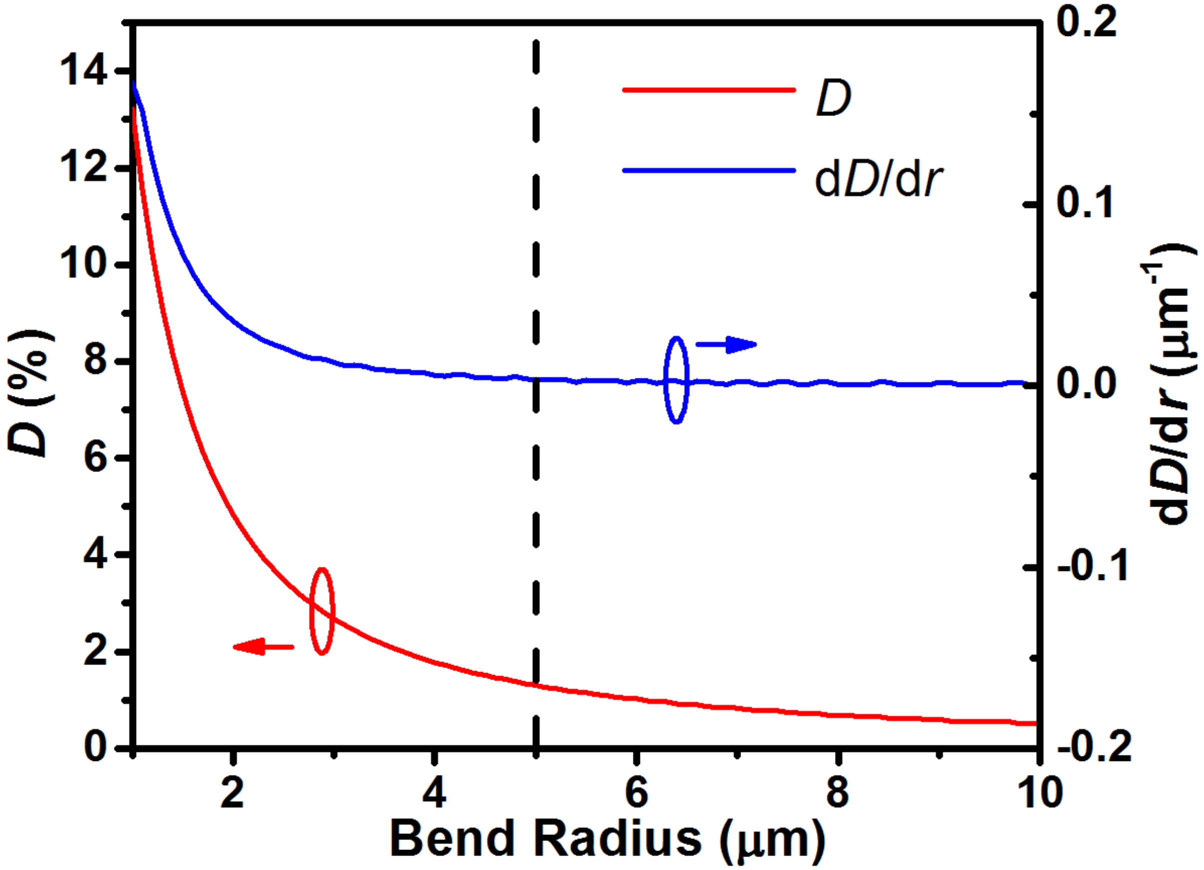

Supplementary Figure S2. Refractive index distortion *D* (red) and distortion changing rate d*D*/d*r* (blue) as a function of bend radius.

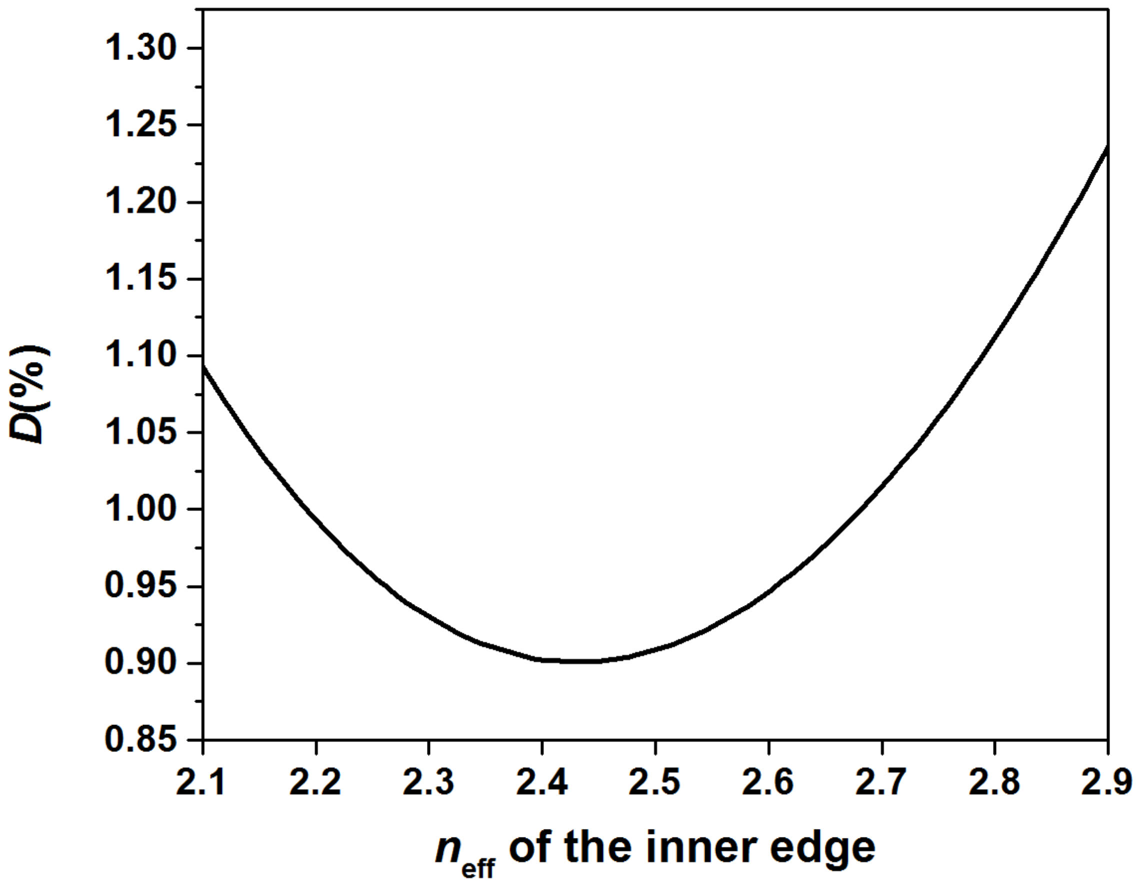

Supplementary Figure S3. Refractive index distortion *D* versus effective refractive index of inner edge. To validate the aforementioned linear approximation, we keep the effective refractive index of the outer edge ($\rho=r_2$) at 2.20 and scan the effective refractive index of the inner edge. When the effective refractive index of the inner edge equals 2.435, D is minimized. The value is very close to 2.43 that calculated from Eq. (4).

Supplementary Note S1.

It is very challenging to fabricate the trapezoidal shape pillars precisely. Fabricated silicon pillars have round corners and curved sidewalls due to electron diffraction and charge accumulation. Pre-compensation has been made in the E-beam lithography pattern to ensure the desired pattern can be precisely transferred into silicon layer. To quantitatively evaluate the pre-compensation, we define a pattern transfer fidelity factor (PTFF) as:

$$PTFF = \frac{\oiint A_d A_f \, dS}{\oiint A_d^2 dS \oiint A_f^2 dS}$$

Here A_i (i = d for design and i = f for fabricated pattern) is the silicon distribution function, which is defined as:

$$A_i = \begin{cases} 1 & \text{silicon} \\ 0 & \text{non-silicon} \end{cases}$$

Take the following picture for example. A_d =1 in the trapezoid marked in black, and A_f =1 in the bright region (silicon). Fabrication parameters have been carefully tuned to make the PTFF as close to 1 as possible.

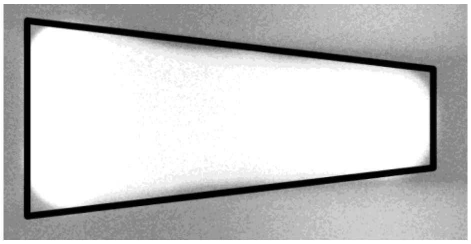

Supplementary Table S1. PTFF of the four types of silicon pillars.

Type	Desired shape	PTFF<br>dS is the resolution of SEM images
Non-tuned	150 nm top base and 150 nm bottom base, 500nm height	0.98
Under-tuned	120 nm top base and 190 nm bottom base, 500nm height	0.97
Optimally tuned	140 nm top base and 210 nm bottom base, 500nm height	0.95
Over-tuned	70 nm top base and 210 nm bottom base, 500nm height	0.98

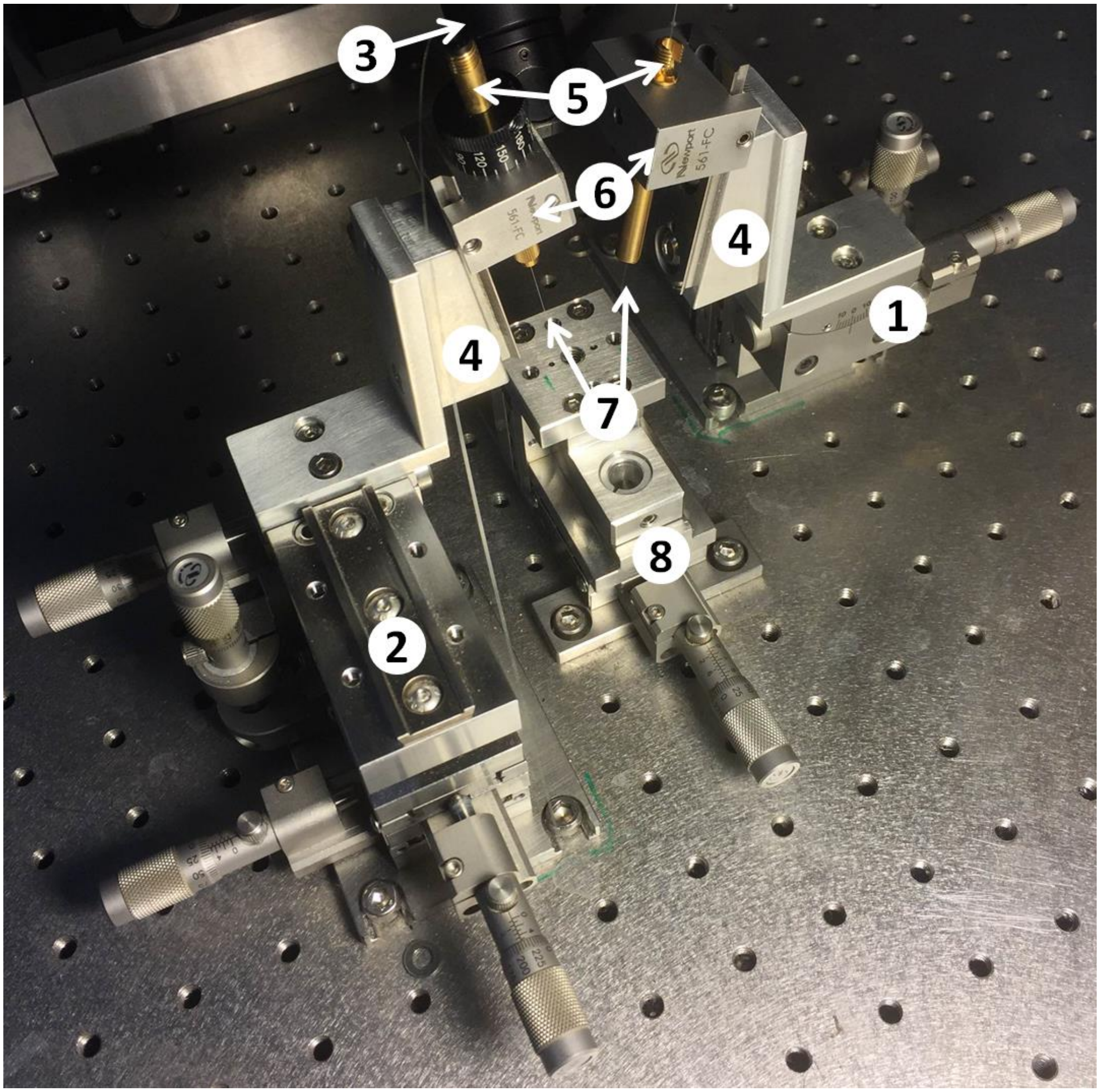

Supplementary Figure S4. Grating coupler testing set-up. (1) Tilting stage (2) xyz stage (3) 45° tilted camera (4) 10° wedges (5) fiber chuck (6) fiber chuck holder (7) fiber (8) sample stage

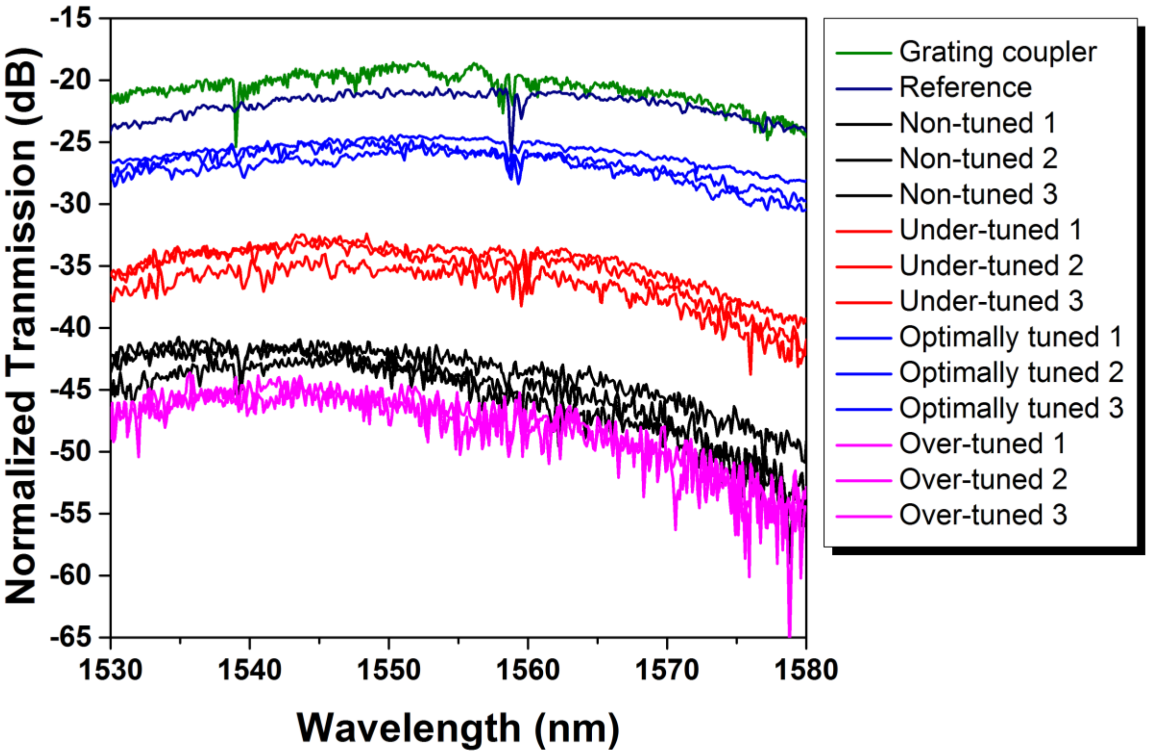

Supplementary Figure S5. Transmission spectra of all devices without subtracting grating couplers' response. The abnormal spikes in the transmission spectra are caused by the imperfect and inconsistent fabrication of grating couplers.

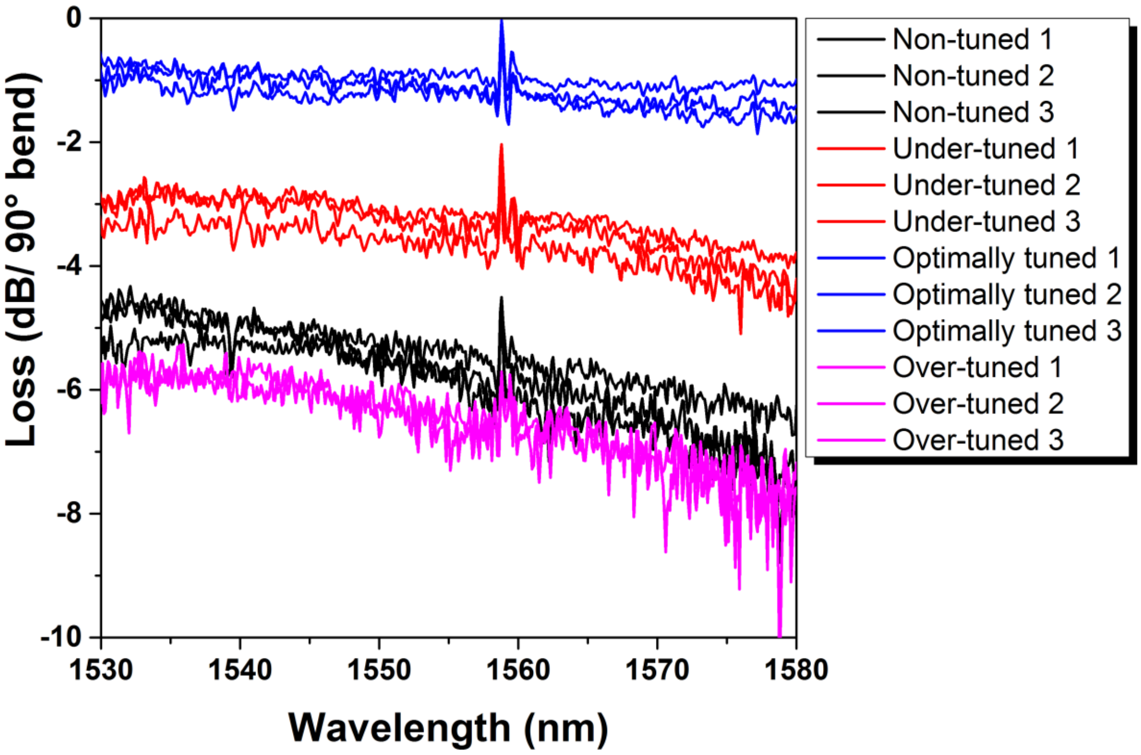

Supplementary Figure S6. Transmission spectra of all devices after subtracting grating couplers' response. The abnormal spikes in the transmission spectra are caused by the imperfect and inconsistent fabrication of grating couplers.

Supplementary Table S2. Simulation results for SWG waveguide bends with different duty cycles. The simulated losses of the optimally tuned SWG waveguide bends with different duty cycles are summarized in the table below. The tuning strength $|T|^2$ decreases when duty cycle δ increases because the mode delocalization induced loss reduces as the average refractive index increases. When δ reaches 0.7, the rectangular shape is the optimized design. One should notice that rectangular shape SWG bend also has a pre-compensated index profiles as the difference of arc length of the inner and outer edges creates gradient refractive index distributions along the radial direction of the bend. For δ>0.7, as the average refractive index of the SWG waveguide is high enough to prevent eminent mode delocalization, the geometrical tuning is unnecessary.

Duty cycle δ	Loss(dB) per 90° bend rectangular silicon pillars	Tuning factor of optimized trapezoidal silicon pillars	Loss(dB) per 90° bend trapezoidal silicon pillars	Loss reduced
0.3	6.098	$T=-0.111+i1.333$, $\|T\|^2=1.790$	1.130	81.5%
0.4	1.796	$T=-0.083+i0.583$, $\|T\|^2=0.347$	0.449	74.9%
0.5	0.383	$T=-0.067+i0.400$, $\|T\|^2=0.164$	0.192	49.9%
0.6	0.143	$T=-0.333+i0.028$, $\|T\|^2=0.112$	0.128	10.5%
0.7	0.069	$T=0$, $\|T\|^2=0$	NA	NA
0.8	0.104	$T=0$, $\|T\|^2=0$	NA	NA
